# Deaths from COVID-19 among workers hospitalized at Hospital Municipal Ronaldo Gazolla from the perspective of work precariousness

**DOI:** 10.47626/1679-4435-2024-1312

**Published:** 2025-08-25

**Authors:** Ana Beatriz dos Santos Domingos, Leonardo do Vale Carvalho Chaves, Aline Silva-Costa, Lúcia Rotenberg

**Affiliations:** 1 Social Work, Hospital Municipal Ronaldo Gazolla, Rio de Janeiro, RJ, Brazil; 2 Department of Epidemiology and Biostatistics, Institute of Collective Health, Universidade Federal Fluminense, Niterói, RJ, Brazil; 3 Laboratory of Education in Environment and Health, Oswaldo Cruz Institute, Fiocruz, Rio de Janeiro, RJ, Brazil

**Keywords:** occupational health, COVID-19, job security, death., saúde do trabalhador, covid-19, precarização do trabalho, óbito.

## Abstract

**Introduction:**

Among the inequalities heightened by the COVID-19 pandemic are those linked to work. Informal workers and the unemployed - already vulnerable before the pandemic - had greater difficulty adopting social distancing. This study analyzes occupational data from patients at a referral hospital for COVID-19 in the city of Rio de Janeiro, Brazil, considering the scarcity of information on workers’ health during the pandemic.

**Objectives:**

This research sought (i) to investigate the relationships between the type of employment relationship and deaths and (ii) to describe the most frequent occupations according to the type of employment relationship and the percentage of deaths.

**Methods:**

One thousand four hundred and four medical records of hospitalizations that occurred between August 2021 and November 2021 were analyzed.

**Results:**

The most represented professions were vendors/sellers, masons/bricklayers, doormen, security guards, drivers, and rideshare drivers. Compared to formal workers, the relative risk of death was 77% higher among homemakers, after adjusting for age. The relative risk of death was 11 and 29% higher among informal workers and the unemployed, respectively, compared to formal workers, but this difference was not statistically significant. Doormen, administrative assistants, salespersons, janitors/custodians, and cleaners had the highest rates of death.

**Conclusions:**

Attention should be given to the increased severity of COVID-19 among homemakers, possibly resulting from an unfavorable health profile in terms of comorbidities in this group.

## Abstract (pt)

**Introdução:**

Entre as desigualdades potencializadas pela pandemia de covid-19, estão aquelas ligadas ao trabalho. Os trabalhadores informais e os desempregados - já vulnerabilizados antes da pandemia - tiveram maior dificuldade de adotar o distanciamento social. Este estudo analisa dados ocupacionais de pacientes de um hospital de referência para a covid-19 no município do Rio de Janeiro, considerando a escassez de informações sobre a saúde dos trabalhadores durante a pandemia.

**Objetivos:**

A pesquisa buscou investigar as relações entre o tipo de vínculo empregatício e os óbitos, além de identificar as ocupações mais frequentes segundo o tipo de vínculo e o percentual de óbitos.

**Métodos:**

Foram analisados 1.404 prontuários de internações ocorridas entre agosto e novembro de 2021.

**Resultados:**

As profissões mais representadas foram: comerciantes, pedreiros, porteiros, seguranças/vigilantes, motoristas e motoristas de aplicativo. Em comparação às trabalhadoras formais, o risco relativo de óbito foi 77% maior entre as trabalhadoras “do lar”, após ajuste pela idade. O risco relativo de óbito foi 11 e 29% maior entre os trabalhadores informais e os desempregados, respectivamente, em comparação aos trabalhadores formais, porém sem significância estatística. Os porteiros, auxiliares administrativos, vendedores, auxiliares de serviços gerais e domésticas apresentaram os maiores percentuais de óbito.

**Conclusões:**

Merece atenção o maior agravamento da covid-19 entre as trabalhadoras “do lar”, possivelmente decorrente de um quadro desfavorável em termos de comorbidades neste grupo.

## INTRODUCTION

The COVID-19 pandemic represented a health crisis affecting various areas of life, having involved “environmental, economic, social, cultural, and political processes and their inextricable interdependencies”.^1^ The Brazilian labor market was severely impacted, with 2.7 million people away from work due to social distancing measures as of September 2020; 14.4 million people were out of work across the country and 15.3 were not seeking employment at all, whether due to the pandemic or to a lack of jobs.^2^ Informal workers were most affected.^3^

Analyses on morbidity and mortality due to COVID-19 have highlighted unemployment and poor socioeconomic and health conditions as drivers of health inequity.^4^ Part of the population did not adhere to social distancing, either due to the need to preserve some source of income or due to housing conditions.^5^ Social distancing is often infeasible when homes are in close proximity, lack sufficient ventilation, or are overcrowded (too many people living in the same room), increasing the risk of contracting COVID-19.^6^ It bears stressing that, according to a Nassif-Pires et al.^7^ study of Brazilian National Health Survey data, when infected by the SARS-CoV-2 virus, the poorest are most likely to have unfavorable outcomes, given their higher prevalence of comorbidities and more precarious access to health care. In short, socioeconomically disadvantaged groups tend not only to have increased exposure to the virus^8^ but also to have greater odds of negative outcomes once infected.^7^

The social protections guaranteed by formal employment are another aspect of inequalities regarding COVID-19. Formal workers who required more than 15 days’ leave of absence due to COVID-19, for example, were able to apply for a temporary disability benefit from Social Security, which was not the case for informal workers. This is a key aspect, as there is no situation in which Social Security could be more important than in the context of a pandemic.^9^ The COVID-19 pandemic served to compound inequalities at a time when workers in Brazil were already facing a buildup of significant rollbacks of labor rights and social security protections.^10^ Fernandes^11^ analyzed the increase in precarious work during the pandemic, defining this concept as a multidimensional construct that encompasses

“(1) unstable employment relationships, resulting from insecure hiring, temporary contracts, involuntary part-time work, outsourcing; (2) inadequate and unstable income; and (3) insufficient rights and protections, with reduced collective representation of workers, which entails reductions in workers’ power to react to degrading conditions, lack of social security, and rollbacks in regulatory support for occupational safety” [free translation].

Type of occupation is another relevant factor implicated in morbidity and mortality due to COVID-19. As noted by Santos et al.,^5^ knowledge about workers’ health during the pandemic has focused mainly on health care providers, as they had the most contact with afflicted patients. Little is known about the health of workers outside the health care sector. The authors highlighted the invisibility of so-called essential workers during the pandemic, such as sanitation workers, motorcycle couriers, and delivery drivers.^5^

Furthermore, in Brazil, there are major barriers to epidemiological studies of workers’ health, due to underreporting of occupation in health information systems.^5,12^ For example, calculation of the frequency of completeness of the profession/occupation field in the Brazilian Mortality Information System (*Sistema de Informação de Mortalidade*, SIM) found that 97.73% of reported COVID-19 deaths lacked this information.^12^ Specifically regarding the COVID-19 pandemic, morbidity statistics do not record information on occupation; therefore, they preclude any assessment of potential risks to certain groups of workers, or detection of focal spread of the disease in association with occupational activities.^13^

Considering the scarcity of information on workers’ health during the pandemic, and since informal workers and the unemployed already constituted a vulnerable group before the pandemic, the present study sought to assess occupational data from patients hospitalized with COVID-19 at a referral center in the city of Rio de Janeiro. Our hypothesis was that informal workers, as well as the unemployed, would be at greater risk of death when compared to formal workers. Secondarily, we sought to identify the most frequently represented occupations in the study sample.

The study aimed to (i) investigate potential associations between the type of employment relationship and death, as well as (ii) describe the most frequent occupations in the sample according to the type of employment relationship and the percentage of deaths.

## METHODS

The study was carried out at Hospital Municipal Ronaldo Gazolla (HMRG), popularly known as “Hospital de Acari” after the neighborhood in which it is located, in Rio de Janeiro, Brazil. HMRG has been in operation since March 2008 and is classified as a medium-complexity facility. On March 23, 2020, it was designated exclusively for the treatment of COVID-19 patients, serving as a referral hospital for COVID-19 in the city of Rio de Janeiro. By 2021, HMRG had the largest intensive care unit (ICU) in Latin America dedicated exclusively to COVID-19 patients. It admitted a total of 15,355 such patients between March 2020 and November 2021, when the last patient was discharged.

The data used in this study were all obtained from the HMRG electronic medical record, based on information provided by the hospital’s Social Services department. At the initiative of one of the authors, a social worker at HMRG, authorization was given-starting in August 2021-to include a set of questions in the electronic medical record in order to meet the objectives of the present study.

Medical records corresponding to patients admitted between August 2021 and November 2021 cover a set of 3,033 hospitalizations of patients aged between 18 and 104 years, which constitute the baseline data universe available for the study. Of these records, 1,538 (50.7%) corresponded to male patients and 1,495 (49.3%) to female patients; in total, there were 1,488 hospital discharges, 1,451 deaths, 17 discharges against medical advice, and 77 transfers to other health facilities. This corresponds to a discharge rate of 50.6% and a mortality rate of 49.4%, after excluding patients who left against medical advice and those transferred to other facilities.

It is worth noting that the study covers a milder period of the pandemic compared to its earlier wave of 2020. The following parameters were available to the investigators: (i) patient’s sex, age, income, and household status as beneficiary of social/income transfer programs; (ii) patient’s occupation and type of employment relationship; and (iii) data on hospital discharge or death.

The exclusion criteria were as follows: (i) patients aged 71 or older; (ii) students, as reported in the “occupation” field; (iii) retirees, as reported in the “occupation” field; (iv) patients who left against medical advice or were transferred to other facilities, given the impossibility of determining whether the patient was ultimately discharged or died; and (v) patients missing data for the “employment relationship” variable. A total of 1,989 medical records were excluded, for a final sample of 1,044 medical records available for analysis (except regarding occupation, which was only included in 603 medical records).

Patients were stratified into three groups by employment relationship: formal workers, informal workers, and the unemployed. Formal workers were defined as those with an employment contract regulated by the Brazilian Consolidation of Labor Laws (*Consolidação das Leis do Trabalho*, CLT), whether they were hired directly by their employer or via a third-party company. Informal workers included those gainfully employed in a company or working from home without a contract or in an unregulated occupation, those who classified themselves as self-employed, and “individual microentrepreneurs” (*microempreendedores individuais*, MEI).

These three distinct employment situations were pooled into a single category because we encountered ambiguity in the classification of data from medical records when considering informal workers, selfemployed workers, and MEI as separate categories. For example, rideshare drivers could classify themselves as informal workers or self-employed, while workers who were formally in the MEI category could classify themselves as self-employed. A subgroup analysis of these three categories found no difference in the relative risk (RR) of death for each group separately as compared to formal workers, which further supported our decision to treat them as a single pooled category, which we termed “informal workers”. For the purposes of this study, the unemployed were defined as people who were not working but were available and actively looking for work.

In addition to these groups, there was a contingent of 44% of the female population whose “occupation” variable was listed as “homemaker”, a term generally associated with unpaid domestic work. Although unpaid domestic work is considered by the Brazilian Institute of Geography and Statistics (IBGE) as “economic inactivity” for the purposes of quantifying the economically active population, this group was included in our analyses, considering that many of these women combine domestic work with gainful activities (direct sales, etc.).^14^ After the decision was made to include this group in the analyses, the variable “employment relationship” was renamed “worker category”, to cover four types of workers: those with a formal employment relationship, informal workers, the unemployed, and “homemakers”.

Data for the “occupation” variable were filled in freely according to the information provided by the patient or next of kin and were categorized according to the Brazilian Classification of Occupations (*Classificação Brasileira de Ocupações*, CBO), a system for classifying and coding occupational activities used widely in Brazil since its creation by the Ministry of Labor and Employment in 1994. Its objective is to standardize and organize information on all occupations in the country, to facilitate identification and analysis of data on the labor market.

Occupations are classified according to the activities involved, considering the necessary set of skills and competencies, the required training, and other workrelated characteristics. The baseline classification considers 10 occupational groups. From this more generic categorization, occupations are further classified in increasingly granular detail, with each occupation ascribed a unique numeric code of five or six digits which corresponds to a detailed description of its activities and requirements.

Once all occupations had been classified individually, similar professions were pooled together, such as “driver”, “company driver”, “bus driver,” or “school transport driver”, which were all categorized as “driver”.

The data was entered into Microsoft Excel spreadsheets. Data processing was performed in the SPSS statistical package, version 23. Initially, sociodemographic profile, social benefits, worker category, outcome (discharge vs. death), and the most frequent occupations in the sample were described. Next, the most frequent occupations were listed by type of employment relationship. Data for these occupations were presented according to the percentage of deaths, with information on age and type of employment relationship.

The percentage of deaths was calculated considering the number of deaths observed in each occupation in relation to the number of hospitalized patients who reported that particular occupation. Finally, to test for association between worker category and mortality, the RR of death was estimated via a Poisson model with robust variance, considering 95% CIs and adjustment for sex and age. As the “homemaker” category included only female subjects, women with a formal employment relationship were considered as the reference category for this group for all analyses, so that comparisons would be made between groups homogeneous in terms of sex.

This study is nested within the research project *Perfil dos trabalhadores acometidos pela covid-19 - o enfoque da Seguridade Social num contexto de precarização do trabalho* [Profile of workers affected by covid-19: a Social Security lens in a context of precarious work], which was approved by the Research Ethics Committees of the Rio de Janeiro Municipal Health Department (opinion no. 5,680,286; Certificate of Submission for Ethical Appraisal: 60687022.6.3001.5279) and the Sérgio Arouca National School of Public Health/Oswaldo Cruz Foundation (ENSP/FIOCRUZ) (opinion no. 5,592,613; Certificate of Submission for Ethical Appraisal: 60687022.6.0000.5240).

## RESULTS

Men made up the majority of the study population (55.4%), and the age group 50 or older made up the largest number of subjects. Around half of subjects reported an income of up to 1.5 times the minimum wage, which corresponded to approximately BRL 1,650.00 at the time of the study. Informal workers constituted the largest category (n = 389), followed by those with a formal employment relationship (n = 279). Approximately 60% of the sample was discharged from hospital. The occupations most represented in the group were vendor/seller, mason/bricklayer, doorman, security guard, driver, rideshare driver, and janitor/custodian. Overall, 77% of workers did not receive any social benefits. *Auxílio Brasil* (its name at the time of the survey) was the most common social benefit across the group, followed by retirement and *Auxílio Emergencial* (a COVID-19-specific benefit, described in further detail below) (Table 1).

**Table 1:** Sociodemographic and occupational profile versus social benefits and recovery in a sample of workers hospitalized due to COVID-19 at Hospital Municipal Ronaldo Gazolla from August 2021 to November 2021 (n = 1,044)

Variable	n	%
Sex		
Male	578	554
Female	466	44.6
Age range (years)		
18-30	98	94
31-50	376	36.1
>50	570	54.5
Income^*^		
None or precarious	252	27.5
Up to 1.5x minimum wage	423	46.2
2x minimum wage or more	241	26.3
Recovery		
Discharge	621	59.5
Death	423	40.5
Worker category		
Formal	279	26.7
Informal	389	37.3
Unemployed	179	17.1
Homemaker	197	18.9
Most common occupations		
Vendor/seller	30	3.18
Mason/bricklayer	26	2.95
Doorman	25	2.95
Security guard	24	2.83
Driver	24	2.76
Rideshare driver	23	2.72
Janitor/custodian	22	2.60
Domestic worker	20	2.36
Cook	19	2.24
Salesperson	19	2.24
Caregiver	16	1.89
Attendant/clerk	13	1.53
Barber/hairdresser	13	1.53
Administrative assistant	12	1.42
Mechanic	10	1.18
Cleaner	9	1.06
Social benefits^*^		
None	707	77.00
*Auxílio Brasil*	63	6.9
Retirement	59	6.4
*Auxílio Emergencial*	43	4.7
Pension	31	3.4
*Benefício de Prestação Continuada*	9	1.00
Unemployment benefits	3	0.3
Temporary disability insurance (sickness benefits)	3	0.3

* Missing data.

Professions included in CBO Occupational Group 5 (Service workers and salespersons at shops, stores, and markets) encompassed the largest number of subjects (255 workers). This was followed by Occupational Group 7 (Workers in the production of industrial goods and services) with 144 subjects, and Occupational Group 3 (Mid-level technicians), with 47 subjects (not shown in the table).

Table 2 describes the sociodemographic data relative to social benefits and discharge versus death stratified by worker category. There was a male predominance in all categories, except for the “homemaker” category which, by definition, was made up exclusively of women. Homemakers were older, with 80.2% in the over-50 age group, a much higher percentage than that observed in the other groups. Around 60% of workers with a formal employment contract reported an income of up to 1.5 times the minimum wage, which corresponded to approximately BRL 1,650.00 at the time of the study. The proportion of workers earning two or more times the minimum wage was similar among those with and without formal employment contracts. In all categories, the majority of subjects did not receive any social benefits, with percentages ranging from approximately 60% to approximately 87%; the lowest percentage was found among homemakers (41.1%). Regarding deaths, the lowest percentage was observed among formal workers (31.2%), with mortality rates of 37.2%, 40.8%, and 59.9% among informal workers, the unemployed, and homemakers, respectively (Table 2).

**Table 2:** Sociodemographic data, social benefits, and recovery in a sample of workers hospitalized due to COVID-19 at Hospital Municipal Ronaldo Gazolla from August 2021 to November 2021 (n = 1,044), stratified by worker category, Rio de Janeiro, 2023

Variable		Formal (n = 279) n (%)		Informal (n = 389) n (%)		Unemployed (n= 179) n (%)	Homemaker (n = 197) n (%)		
Sex Male		186 (66.7)		272 (69.9)		117 (65.4)	0.0		
Female		93 (33.3)		117 (30.1)		62 (34.6)	197 (100.0)		
	Age range (years)								
	18-30		35 (12.5)		29 (7.5)	28 (15.6)		6 (3.0)	
	31-50		130 (46.6)		151 (38.8)	62 (34.6)		33 (16.8)	
	> 50 Income		114 (40.9)		209 (53.7)	89 (49.7)		158 (80.2)	
	2× minimum wage or more		106 (40.9)		113 (34.8)	6 (3.5)		16 (10.0)	
	Up to 1.5× minimum wage		153 (59.1)		140 (43.1)	41 (23.8)		89 (55.6)	
	None or precarious Social benefits		0.0		72 (22.2)	125 (72.7)		55 (34.4)	
	None		242 (86.7)		277 (71.4)	107 (59.8)		81 (41.1)	
	*Auxílio Brasil*		0.0		17 (4.4)	22 (12.3)		24 (12.2)	
	*Auxílio Emergencial*		0.0		14 (3.6)	17 (9.5)		12 (6.1)	
	Retirement		16 (5.7)		28 (7.2)	0.0		15 (7.6)	
	Pension		0.0		3 (0.8)	2 (1.1)		26 (13.2)	
	*Benefício de Prestação Continuada*		0.0		2 (0.5)	1 (0.5)		6 (3.0)	
	Temporary disability insurance (sickness benefits)		3 (1.1)		0.0	0.0		0.0	
	Unemployment benefits Recovery		0.0		1 (0.3)	2 (1.1)		0.0	
	Discharge		192 (68.8)		244 (62.7)	106 (59.2)		79 (40.1)	
	Death		87 (31.2)		145 (37.3)	73 (40.8)		118 (59.9)	

After adjustments, the RR for death was higher among homemakers (RR = 1.77; CI 1.12-2.78), compared to formal workers. The 11% and 29% higher RR of death among informal workers and the unemployed, respectively, was not statistically significant (Table 3).

**Table 3:** Association between worker category and death in a sample of workers hospitalized due to COVID-19 at Hospital Municipal Ronaldo Gazolla from August 2021 to November 2021 (n = 1,044), Rio de Janeiro, 2023

Death	Informal^*^ RR (95%CI)	Unemployed^*^ RR (95%CI)	Homemakers† RR (95%CI)
Crude model	1.20 (0.92-1.56)	1.31 (0.96-1.79)	2.12 (1.39-3.25)
Adjusted model^‡^	1.11 (0.85-1.45)	1.29 (0.95-1.77)	1.77 (1.12-2.78)

^*^Reference category: workers with formal employment contracts.

† Reference category: female workers with formal employment contracts.

‡ For age and sex.

A description of occupations according to the type of employment relationship is given in Table 1. The occupational categories most represented among workers with formal employment contracts were security guard, doorman, janitor/custodian, driver, cook, administrative assistant, and salesperson. Among informal workers, vendors/sellers were the largest category, followed by rideshare driver, mason/ bricklayer, barber/hairdresser, caregiver, taxi driver, and domestic worker. Doorman, janitor/custodian, and mason/bricklayer were the most common categories among the unemployed (Chart 1).

**Chart 1:** Most common occupations in a sample of workers hospitalized due to COVID-19 at Hospital Municipal Ronaldo Gazolla from August 2021 to November 2021 (n = 603), stratified by type of employment relationship

Type of employment relationship	Occupations
Formal	Security guard (n = 20), doorman (n = 19), janitor/custodian (n = 16), driver (n = 12), cook (n = 10), administrative assistant (n = 11), salesperson (n = 11), attendant/clerk (n = 9), nursing technician (n = 8), domestic worker (n = 7)
Informal	Vendor/seller (n = 30), rideshare driver (n = 22), mason/bricklayer (n = 20), barber/hairdresser (n = 13), taxi driver (n = 11), caregiver (n = 12), domestic worker (n = 10), cleaner (n = 8), salesperson (n = 6), driver (n = 8), cook (n = 5)
Unemployed	Doorman (n = 6), janitor/custodian (n = 5), mason/bricklayer (n = 5), cook (n = 4), driver (n = 4), teacher (n = 3), domestic worker (n = 3), hod carrier (n = 3)

n = number of workers in each occupation.

Table 4 lists the most common occupations in the sample, in order of mortality rate, considering hospitalized patients. Doormen and administrative assistants had the highest proportion of deaths, namely 50%, followed by salespersons (42.1%), janitors/custodians (40.9%), and domestic workers (40%) (Table 4).

**Table 4:** Most common occupations in a sample of workers hospitalized due to COVID-19 at Hospital Municipal Ronaldo Gazolla from August 2021 to November 2021 (n = 847), in order of mortality rate among patients for whom information on age and type of employment relationship was available; Rio de Janeiro, 2023

Occupation	Deaths in proportion to hospitalizations (%)	Age (years) mean (SD)	Formal (n)	Informal (n)	Unemployed (n)
Doorman	500	52.7 (10.9)	19	-	6
Administrative assistant	500	45.5 (15.2)	11	-	1
Salesperson	42.1	43.8 (15.3)	11	6	2
Janitor/custodian	40.9	45.9 (14.6)	16	1	5
Domestic worker	40.0	52.4 (9.6)	7	10	3
Driver	39.1	51.9 (10.7)	12	8	4
Barber/hairdresser	38.4	45.1 (10.8)	-	13	-
Attendant/clerk	38.4	39.0 (11.9)	9	3	1
Security guard	37.5	48.6 (10.2)	20	2	2
Mason/bricklayer	34.1	50.8 (9.6)	1	20	5
Caregiver	31.2	54.7 (9.6)	2	12	2
Vendor/seller	30.0	47.3 (12.1)	-	30	-
Cook	31.6	47.2 (11.7)	10	5	4
Mechanic	30.0	49.5 (8.4)	6	4	-
Cleaner	22.2	54.7 (11.6)	1	8	-
Rideshare driver	13.0	46.0 (8.7)	0	22	1

SD = standard deviation.

## DISCUSSION

This study covered workers hospitalized for COVID-19 between August 2021 and November 2021, with a focus on worker category and occupation. As expected for a public hospital, the sample was made up of patients from the lowest income brackets (around 50% reported an income of up to one and a half times the minimum wage), who, therefore, were already vulnerable prior to the pandemic.^1^

Our findings do not support the hypothesis of a higher RR of death among informal workers and the unemployed than in formal workers; however, this may reflect the underlying profile of the sample. These were seriously ill patients, to the point of requiring hospitalization at a time when part of the population had already been vaccinated.^15^ The serious condition of these patients may have contributed to reducing the relevance of socioeconomic factors that would otherwise predispose to negative outcomes among those most vulnerable-in this case, informal workers and the unemployed. Furthermore, the socioeconomic condition of the sample possibly did not exhibit much variability, as it was composed of an already very vulnerable population from a socioeconomic standpoint.

The study showed a 77% increase in the RR of death among homemakers compared to formal workers, with a mortality rate of nearly 60%. The term “homemaker” is often used among the working classes to refer to women who lack formal employment^16^; many combine domestic work with another activity, such as direct sales.^14^

As little is known specifically about “homemakers”, some data can be inferred from studies on so-called “housewives”. Comparative data on the health of gainfully employed women and that of “housewives” can contribute to the discussion on the higher RR of death from COVID-19 among “homemakers”. A study of more than 7,000 women aged 45 to 64 found a higher risk of coronary heart disease and ischemic stroke among “housewives” compared to those who had gainful employment, with the risk being higher among those with lower educational attainment.^17^ These findings corroborate those of Reviere & Eberstein,^18^ who found a higher risk of coronary heart disease among “housewives”. Along the same lines, research on a representative sample of the population of England and Wales also identified a higher risk of all-cause mortality among “housewives” compared to paid workers.^19^

In Brazil, Senicato et al.^20^ reported a greater impact of the illness-wellness continuum on functionality and well-being among housewives. Taken together, these studies suggest that the higher RR of death among “homemakers” is due to their more unfavorable condition regarding pre-existing comorbidities that would act as a risk factor for worse COVID-19 outcomes. Considering that age is a relevant factor in COVID-19 and that this group was older than that of formal workers, the possible residual influence of age in the regression analysis cannot be ruled out, even after adjusting for age.

Sellers/vendors accounted for the largest number of workers, followed by masons/bricklayers, doormen, security guards, drivers, rideshare drivers, and janitors/ custodians. Many jobs have been lost in the trade sector, especially among retail employees. However, according to the 2020 Annual Trade Survey (*Pesquisa Anual de Comércio*, PAC) published by IBGE, two sectors involved in activities considered essential during the pandemic-hypermarkets/supermarkets and drugs/ cosmetics-saw an increase in staffing, increasing the risk of contamination among their workers.

Social distancing led to increased demand for delivery services and ride-hailing services.^21^ The contingent of workers employed in these activities increased by approximately 42% from the 1st quarter of 2016 to the 1st quarter of 2020.^22^ Whether because an occupation involves contact with many people (such as vendors/sellers, drivers, and doormen) or because of the risks arising from the use of public transport (as in the case of domestic workers^23^), contracting COVID-19 was an occupational hazard for many professional categories.

However, the occupations most represented in our sample did not necessarily correspond to mortality rates. For example, vendors/sellers constituted the largest profession in the sample overall, but their mortality rate-30%-was comparatively low. Conversely, administrative assistants had a mortality rate of 50%, without being a particularly large group compared to other occupations. This is consistent with the observation by Santos et al.^5^ that one’s risk of contracting COVID-19 does not necessarily correspond to one’s risk of death, probably due to determinants other than exposure to the virus. As we refer to the percentage of deaths in a sample of hospitalized patients, a high proportion in a given occupation does not necessarily mean this was the profession most affected by COVID-19 in the population.

Occupations with the highest percentages of deaths included doormen, administrative assistants, salespersons, janitors/custodians, domestic workers, drivers, barbers/hairdressers, attendants/clerks, and security guards, all with mortality above 35%. Galindo et al.^24^ analyzed the occupations most affected by COVID-19 fatalities in 2020 based on different databases, all restricted to formal workers.

Of the five most affected occupations-truck driver (regional and international routes), security guard, building doorman, assembly line feeder, and cleaner- some corresponded to occupations also recorded in out sample, such as doormen (50% mortality) and drivers (39.1%). Mortality among doormen and drivers was 3.5 times higher than the population average; these are activities that require social contact and involve circulation among many persons, were unlikely to be interrupted during the pandemic, and offer no possibility of remote work.^24^

Another study by the Institute of Applied Economic Research (IPEA; published as Technical Note No. 76), based on the Rio de Janeiro State Health Department database, found that the odds of dying among security workers in 2020 were 2.25 times higher than those of persons employed in other activities.^25^ It is clear from our data that workers who were unable to curtail their mobility were most affected.

Decree No. 10,282, issued on March 20, 2020,^10^ defined “essential” public services and activities as those involved in health care, social service, care for vulnerable populations, and public and private security and transportation (intermunicipal, interstate, and international passenger transport), among others. Essential workers were more exposed to the twin risks of contamination and death. It is worth noting that both IPEA surveys mentioned above dealt exclusively with formal workers, since their databases are based, among other sources, on the Annual Social Information Report (*Relação Anual de Informações Sociais*, RAIS), a record of employment relationships obtained directly from employers and, therefore, refer to a specific group of the Brazilian population.^5^

The high rate of deaths among administrative assistants may have resulted from customer-facing activities, since, according to the CBO, this occupation is involved in providing support services in various areas (human resources, administration, finance, logistics) and assisting suppliers and clients in the public or private sectors, under formal (CLT) employment contracts. Domestic workers also had a high percentage of deaths. Using data from the 2019 Continuous National Household Sample Survey (*Pesquisa Nacional por Amostras de Domicílios*, PNAD), Pinheiro et al.^26^ analyzed the vulnerability of domestic workers-a contingent of more than 6 million in Brazil, the majority of whom are Black women.

Despite the efforts of the Labor Prosecutor’s Office to safeguard the activities of this group (Technical Note 04/2020),^27^ such as by ensuring access to personal protective equipment and alternative working hours (to avoid rush hour on public transport), domestic workers were nonetheless greatly affected by the pandemic, and can be said to be part of the group of essential workers who were largely invisible in statistics regarding the impact of COVID-19.^5^

The data collected for the present study allowed us to analyze our sample regarding social benefits, including *Auxílio Emergencial* (Emergency Aid), an emergency cash transfer-type benefit implemented as public policy during the COVID-19 pandemic.^28^ This measure sought to mitigate the socioeconomic effects of COVID-19, especially on informal workers, the self-employed, the unemployed, small retail business owners, and domestic workers. However, only around 10% of the unemployed and 4% of informal workers in our sample reported receiving this benefit. These low percentages possibly reflect the difficulties faced by the population in actually accessing the benefit, which included issues with the Taxpayer Identification Number through which the benefit is granted, long delays in the decision to make payment, difficulties in accessing and using digital technologies by the most vulnerable groups, and inefficiencies in distribution, as the benefit is known to not have reached all those who needed it.^29^

The secondary database that formed the basis of this study placed significant limitations on our research, as the electronic medical records were filled out by hospital social workers, some of whom may not have been aware of the importance of completing specific fields, especially those corresponding to occupational data. Furthermore, fields relating to housing conditions were left mostly unfilled, which led us to exclude this information from our final database.

Another limitation is that our analyses refer solely to inpatients admitted to a public hospital during a specific phase of the pandemic (August to November 2021), which was lower in acuity than the initial wave in 2020 and by which time part of the Brazilian population had already been vaccinated.^15^ Therefore, our findings cannot be generalized to the overall population, nor to analyses of hospitalizations and deaths of workers during other stages or waves of the pandemic.

These limitations notwithstanding, the database allowed us to extract reliable information about hospitalization and recovery from COVID-19 in a population that was vulnerable at the outset, even before the pandemic struck. Considering the scarcity of data on workers’ health during the pandemic, the inclusion of occupational data in the electronic medical record of such a large hospital is, in itself, an expression of the contribution of this study to the field of occupational health. This allowed us to work with a dataset quite distinct from that usually addressed in the literature on social inequalities, in which occupational data are rarely discussed; this is particularly important given underreporting of the “occupation” variable in official databases in Brazil.

Another merit of this study concerns the inclusion of so-called homemakers. Given the unique characteristics of unpaid domestic work, in principle it would not be appropriate to include homemakers or housewives as a category in the “employment relationship” variable, nor in the “occupation” variable. The lack of access to information on pre-existing conditions prevented us from testing whether the higher RR of death among “homemakers” was due to their more unfavorable condition in terms of comorbidities that would act as a risk factor for worse COVID-19 outcomes. Despite these limitations, inclusion of this group was a net positive in the sense that it generated knowledge about a population that is neglected both in the occupational health literature and in official statistics, due to the classification of homemakers as economically inactive.

## CONCLUSIONS

Our empirical data on workers hospitalized due to COVID-19 at a specific stage of the pandemic do not support the hypothesis of a greater risk of death among groups known to be vulnerable, such as informal workers and the unemployed. Conversely, homemakers had a high mortality rate and a higher RR of death than formal workers, possibly due to their more unfavorable condition in terms of pre-existing comorbidities.

Regarding occupations, our data shed light on occupations that have previously received little visibility from the point of view of COVID-19 mortality. Reflecting on work-related inequalities during the COVID-19 pandemic led us to a particular focus on doormen and domestic workers, who were among the occupations with the highest percentages of deaths. This finding harks back to the first deaths from COVID-19 in the cities of São Paulo and Rio de Janeiro: a building doorman and a domestic worker, respectively.^23^ Both fell ill after coming into contact with patients who had contracted the disease abroad, demonstrating that the first to become infected were not the first to die- which unequivocally reflects the central role of social inequality/inequity as a factor in analyses of COVID-19 and of health in general.

## References

[1] Diniz DS, Teixeira ES, Almeida WGR, Souza MSM. (2021). Covid-19 e doenças negligenciadas ante as desigualdades no
Brasil: uma questão de desenvolvimento
sustentável. Saude Debate.

[2] Agência IBGE Notícias (2020). PNAD COVID19: 3,3% da população ocupada estavam afastados
do trabalho devido ao distanciamento social na quarta semana de
setembro.

[3] Almeida WS, Szwarcwald CL, Malta DC, Barros MBA, Souza PRB, Azevedo LO (2020). Mudanças nas condições
socioeconômicas e de saúde dos brasileiros durante a pandemia
de COVID-19. Rev Bras Epidemiol.

[4] Barreto NMPV, Rios JDC, Ribeiro EB, Torreão PL, Oliveira SAL, Teixeira JRB (2021). Vulnerabilidades sociais relacionadas à
infecção e mortalidade por covid-19: uma revisão
sistemática. Rev Saude Col UEFS.

[5] Santos KOB, Fernandes RCP, Almeida MMC, Miranda SS, Mise YF, Lima MAG. (2020). Trabalho, saúde e vulnerabilidade na pandemia de
COVID-19. Cad Saude Publica.

[6] Rede CoVida (2020). Saúde do Trabalhador: riscos e vulnerabilidades.

[7] Nassif-Pires L, Carvalho L, Rawet E. (2020). Multidimensional inequality and COVID-19 in Brazil; Public Policy Brief
Nº. 153.

[8] Demenech LM, Dumith SC, Vieira MECD, Neiva-Silva L. (2020). Desigualdade econômica e risco de infecção e
morte por COVID-19 no Brasil. Rev Bras Epidemiol.

[9] Berwanger JLW, Buralde LK. (2020). In: Anais do II Congresso Internacional da Rede Ibero-Americana de
Pesquisa em Seguridade Social.

[10] Brasil (2020). Regulamenta a Lei nº 13.979, de 6 de fevereiro de 2020, para definir os
serviços públicos e as atividades essenciais.

[11] Fernandes RCP. (2023). O construto multidimensional trabalho precário, o futuro
do trabalho e a saúde de trabalhadoras(es). Cad Saude Publica.

[12] Santos C, Ferreira VM, Cavalcante JR, Ribeiro PC, Castro HA, Gutierrez AC (2022). O que aprendemos diante dos dados que não temos? O caso da
variável profissão/ocupação nos bancos de
síndrome gripal, síndrome respiratória aguda grave e
mortalidade no Brasil. SciELO Preprints.

[13] Jackson JM, Assunção AÁ, Algranti E, Garcia EG, Saito CA, Maeno M. (2020). A saúde do trabalhador e o enfrentamento da
COVID-19. Rev Bras Saude Ocup.

[14] Meyer AVTL, Oliveira ENP, Coelho RN, Aquino CAB. (2019). Trabalho doméstico e empreendedorismo: a
intensificação laboral das donas-de-casa. R Laborativa.

[15] Barcellos C, Xavier DR. (2022). As diferentes fases, os seus impactos e os desafios da pandemia
de covid-19 no Brasil. Rev Eletron Comun Inf Inov Saude.

[16] Rotenberg S, De Vargas S. (2004). Práticas alimentares e o cuidado da saúde: da
alimentação da criança à
alimentação da família. Rev Bras Saude Matern Infant.

[17] Carson AP, Rose KM, Catellier DJ, Diez-Roux AV, Muntaner C, Wyatt SB. (2009). Employment status, coronary heart disease, and stroke among
women. Ann Epidemiol.

[18] Reviere R, Eberstein IW. (1992). Work, marital status, and heart disease. Health Care Women Int.

[19] Weatherall R, Joshi H, Macran S. (1994). Double burden or double blessing? Employment, motherhood and
mortality in the Longitudinal Study of England and Wales. Soc Sci Med.

[20] Senicato C, Lima MG, Barros MBA. (2016). Ser trabalhadora remunerada ou dona de casa associa-se à
qualidade de vida relacionada à saúde?. Cad Saude Publica.

[21] Graça GMB, Araújo JM. (2020). Coronavírus e uberização: como a pandemia
expôs a vulnerabilidade dos motoristas de aplicativo submetidos a um
regime precário de direitos no Brasil. Rev Esc Jud TRT4.

[22] Manzano M, Krein A. (2020). A pandemia e o trabalho de motoristas e de entregadores por aplicativos
no Brasil.

[23] Pinheiro L, Tokarski CP, Posthuma AC (2021). Entre relações de cuidado e vivências de
vulnerabilidade: dilemas e desafios para o trabalho doméstico e de
cuidados remunerado no Brasil.

[24] Galindo EP, Silva SP, Pedreira JU. (2022). Nota Técnica Nº 27.

[25] De Negri F, Galliez RM, Miranda P, Koeller P, Zucoloto G, Costa J (2020). Nota Técnica Nº 76.

[26] Pinheiro L, Tokarski C, Vasconcelos M. (2020). Nota Técnica Nº 75.

[27] Brasil, Ministério Público do Trabalho, Procuradoria
Geral do Trabalho (2020). Nota Técnica Conjunta 04/2020.

[28] Brasil, Presidência da República, Secretaria-Geral,
Subchefia para Assuntos Jurídicos (2020). Altera a Lei nº 8.742, de 7 de dezembro de 1993.

[29] Marins MT, Rodrigues MN, Silva JML, Silva KCM, Carvalho PL. (2021). Auxílio Emergencial em tempos de pandemia. Soc Estado.

